# Increasing Trend in Hospital Deaths Consistent among Older Decedents in Korea: A Population-based Study Using Death Registration Database, 2001–2014

**DOI:** 10.1186/s12904-017-0269-x

**Published:** 2018-01-11

**Authors:** Tran Thi Xuan Mai, Eunsook Lee, Hyunsoon Cho, Yoon Jung Chang

**Affiliations:** 1 Department of Cancer Control and Population Health, National Cancer Center Graduate School of Cancer Science and Policy, Goyang, Republic of Korea; 2 Center for Breast Cancer, Research Institute and Hospital, National Cancer Center, Goyang, Republic of Korea; 3 Department of Cancer Biomedical Science, National Cancer Center Graduate School of Cancer Science and Policy, Goyang, Republic of Korea; 4 Division of Cancer Registration and Surveillance, National Cancer Control Institute, National Cancer Center, Goyang, Republic of Korea; 5 Hospice and Palliative Care Branch, National Cancer Control Institute, National Cancer Center, Goyang, Republic of Korea

**Keywords:** Place of death, Palliative care, Hospital death, Cancer death, Non-cancer death

## Abstract

**Background:**

With improvement in hospice palliative care services and long-term care, Republic of Korea (hereafter South Korea) has recorded significant changes in places of death (e.g., hospital, home), especially among older adults. Over the last few decades, the most common places of death in South Korea were hospitals. However, Koreans, especially older adults, reportedly prefer to receive terminal care and eventually die at home. This study was conducted to investigate trends in places of death among older Korean adults and factors associated therewith.

**Methods:**

Data were obtained from the Korean Death Registration Database maintained by the National Statistical Office. Decedents who died after the age of 65 years from 2001 to 2014 were included in the analysis. For descriptive analysis, proportions of places of death were analyzed and were used to plot graphs for visualizing trends during 13-year period. Logistic regression model was used to evaluate factors associated with places of death (hospital versus home).

**Results:**

Two million three hundred fifty eight thousand two hundred eleven older adult decedents were included in final analysis. Hospitals were the most common places of death (57.82%), followed by homes (32.12%). Dying at social welfare facilities was rare (2.61%). A gradual increase in hospital deaths (31.38% in 2001 to 75.30% in 2014) and a subsequent decrease in home deaths (60.44% to 15.95% over the same period) were noted. Hospital deaths were more likely for younger patients (ORs 1.28, 95% CI 1.27-1.29), females (ORs 1.28, 95% CI 1.27-1.29), and single/divorced or widowed individuals (ORs 1.77, 1.49 and 1.03 respectively). A higher education level and living in urban areas were strongly associated with a higher likelihood of dying in a hospital.

**Conclusion:**

Over the study period, there was a consistent increasing trend in hospital deaths in South Korea. Trends in place of death and factors associated therewith should be more intensely investigated and monitored. Resources and facilities should be increased to fulfill end-of-life care preferences and the needs of an increasingly older population in South Korea.

## Background

Place of death has been suggested as a key indicator and outcome measure of end-of-life care [1–4]. Distributions of places of death hold important implications in public health planning, policymaking, and resource allocation for effective care [5]. Identifying and fulfilling preferences for place of death also have cost implications [6]. Research has indicated that end-of-life care at hospitals is associated with health expenditures that are two [7] to even three times [8] higher than those for end-of-life care at home or community care settings. Therefore, reducing inappropriate hospital admissions, shortening length of hospital stay, and changing the setting of end-of-life care (from hospital care to community care or home care) have garnered greater focus in policy initiatives in the United States [2, 9] and many European countries, particularly England [8, 10].

Approximately half of decedents expressed preference for a home-based end-of-life and eventually die at their usual place of residence [11–14]. However, hospitals, which are consistently regarded as the least preferred place of death, still remain the most common places of death in many developed countries [15, 16]. Nonetheless, studying trends in where people die and assessment of changes in living arrangements, which are likely to influence death places and types of end-of-life care, are important to developing appropriate approaches and policies regarding end-of-life care. Various studies have been conducted to investigate where people actually die; the majority of said studies have mainly focused on cancer decedents [17]. In South Korea, as the population of older adults is expected to grow dramatically in number due to significant increases in life expectancy and population aging, examining deaths from other chronic conditions, such as dementia related diseases, that are more common among older adults will become more important. For this reason, the present study focused on older adults who died from major chronic conditions, including cancer and other diseases.

In South Korea, a shift in the distribution of deaths across settings was observed between 1992 and 2001, from deaths at home to deaths in a hospital [18]. While hospice and palliative care is still a relatively new concept in South Korea, policies and efforts have been undertaken to improve these services [19], such as an initial palliative care program for cancer patients in 2004. To further improve hospice and palliative care services, reevaluating changes in places of death in South Korea is needed, especially among the elderly.

Using a death registration database, we conducted this study to provide insight into trends in places of death among older adults in South Korea and associations with independent determinants thereof, including cause of death, socio-demographic factors, and residential area. Since cancer ranks among the most popular causes of death in South Korea [20], we focused on comparing places of death between cancer and non-cancer decedents. Results from this study could assist health planners with improving the quality of end-of-life health care in South Korea, particularly that for older adults.

## Methods

### Data source

Data for this study were obtained from a death registration database maintained by the National Statistical Office (NSO) in South Korea. According to the Korea Family Registration Act, all deaths must be reported to the local administrative government within one month thereafter. The NSO then collects data through National Vital Statistics System. In this study, we included data of all decedents who died at age 65 years or older from 2001 to 2014. Death certificate records include the following information: time of registration (day, month, and year), place of residence, sex, time of death (year, month, day, and time), place of death, occupation of decedent, by whom death was diagnosed, marital status, education level, and first and second underlying causes of death according to International Classification of Diseases version 10 (ICD-10) [20].

### Outcome measure

The NSO classifies places of death as home, medical institution or hospital, residential institution, public administrative area, road, commercial service facilities (store, hotel, etc.), industrial area, agricultural area, during hospital transfer, other places, and unknown [20]. We selected the most common places of deaths in South Korea [18] and later re-classified outcome variables into four main categories: home; hospital; social welfare-public facilities (hereafter social welfare facility), including residential institution and public administrative area; and elsewhere.

### Explanatory variables

Explanatory variables included were sex, age at death, marital status and education level. In accordance with our target population, we analyzed data on eight illnesses common among older adults that are relevant to end-of-life care [16]. We also included human immunodeficiency virus infection (HIV) in our analysis. Using ICD-10 classification, causes of death were classified into 10 categories: cancer (C00-C97), dementia (F00-F03), ischemic heart disease (I20-I25), cerebrovascular disease (I60-I69), chronic lower respiratory diseases (J40-J47), Parkinson’s disease (G20), Alzheimer’s disease (G30), HIV (B20-B24), and others.

### Statistical analysis

Time trends in proportions of death at a hospital, home, or social welfare facility were described. Associations between place of death and explanatory variables were evaluated using Pearson’s Chi-squared test. Dependent variable was defined as “hospital death” (1) versus “home death” (0). Due to our interest in hospital death versus home death, deaths at other places were excluded from the regression model. We conducted univariate and multivariate logistic regressions. Crude odds ratio and adjusted odds ratio (ORs and aORs) were reported together with 95% confidence intervals (95% CI). Statistical significance was set at *P*-values <0.05.

## Results

**Fig. 1:**
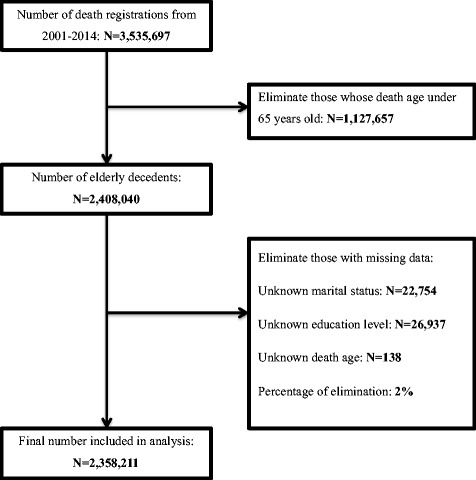
Sample selection process. From 2001 to 2014, 3,535,697 deaths were registered in South Korea. Among those, 2,408,040 deaths were older decedents (age 65 and over). Data was excluded if there was missing in marital status, education level, death age. Percentage of elimination was 2%. Final analysis included 2,358,211 deaths

After excluding 49,829 (2%) subjects with missing data on key variables, the final data set included data on 2,358,211 individuals over the age of 65 years (Table 1, Fig. 1). Overall, 1,090,996 deaths occurred between 2001 and 2007 and 1,267,215 during 2008-2014. Hospitals were the most common place of death (57.8%) over the whole period, followed by death at home (32.1%). Mean age at death was 78.29 years for 2001-2007 and 79.53 years for 2008-2014. The proportion of those who died at 85 years or over increased from 23.5% in 2001-2007 to 28.7% in 2008-2014.

**Table 1:** Characteristics of older decedents from all cases of death, South Korea 2001-2014

Variable	Value	2001-2014		2001-2007		2008-2014	
		*N*	%	*N*	%	*N*	%
All	All	2,358,211	100.0	1,090,996	100.0	1,267,215	100.0
Place of death	Home	757,345	32.1	503,446	46.2	253,899	20.0
	Hospital	1,363,502	57.8	474,251	43.5	889,251	70.2
	Social/Welfare	61,650	2.6	9728	0.9	51,922	4.1
	Others	175,714	7.5	103,571	9.5	72,143	5.7
Age, years	Mean (SD)	78.96	7.99	78.29	7.95	79.53	7.98
	Median ^a^	79	73-85	78	72-84	79	73-85
Age, categorized	65-74	762,012	32.3	385,390	35.3	376,622	29.7
	75-84	976,836	41.4	449,453	41.2	527,383	41.6
	85+	619,363	26.3	256,153	23.5	363,210	28.7
Gender	Male	1,130,903	48.0	510,054	46.8	620,849	49.0
	Female	1,227,308	52.0	580,942	53.3	646,366	51.0
Marital status	Single	32,342	1.4	14,159	1.3	18,183	1.4
	Married	1,034,786	43.9	464,259	42.6	570,527	45.0
	Divorced	55,109	2.3	17,666	1.6	37,443	3.0
	Widowed	1,235,974	52.4	594,912	54.5	641,062	50.6
Education level	Uneducated	948,400	40.2	503,458	46.2	444,942	35.1
	Elementary	826,454	35.1	364,111	33.4	462,343	36.5
	Middle/High school	459,258	19.5	176,074	16.1	283,184	22.4
	University or higher	124,099	5.3	47,353	4.3	76,746	6.1
Residential area	Rural	416,887	21.9	132,609	21.0	284,278	22.4
	City	772,729	40.7	267,865	42.3	504,864	39.8
	Metropolitan	429,049	22.6	139,442	22.0	289,607	22.9
	Capital	281,601	14.8	93,135	14.7	188,466	14.9
Cause of death	Cancer (C00-C97)	588,461	25.0	256,908	23.6	331,553	26.2
	Dementia (F00-F03)	52,341	2.2	23,650	2.2	28,691	2.3
	IHD (I20-I25)	111,048	4.7	52,655	4.8	58,393	4.6
	CVDs (I60-I69)	238,899	10.1	142,146	13.0	96,753	7.6
	COPD (J40-J47)	98,466	4.2	52,177	4.8	46,289	3.7
	Parkinson’s Disease (G20)	21,402	0.9	6417	0.6	14,985	1.2
	Alzheimer’s Disease (G30)	27,005	1.2	7465	0.7	19,540	1.5
	HIV (B20-B24)	163	0.0	30	0.0	133	0.0
	Others	1,220,589	51.8	549,578	50.4	671,011	53.0

^a^Median and interquartile

Overall, hospitals were the most common place of death ranging around 60-70% (Table 2). The proportion of hospital deaths increased with increasing age; the opposite was observed for deaths at home. Hospital deaths accounted for the largest percentage in all age groups, with 63.9% in the 65-74 years age group and 48.2% in the 85+ years age group. Hospital deaths were slightly more frequent among male decedents than female decedents (60.3% vs. 55.6%). Hospital deaths increased with increasing education level (48.9% among uneducated subjects and 72.0% among college/university educated subjects). In terms of residential area, deceased people living in metropolitan areas comprised the highest proportion of hospital deaths, compared to residents in other areas. However, the difference in the proportions of places of death across different urbanization levels was not significant. Results from the Pearson’s chi-squared test were significant for all explanatory variables.

**Table 2:** Characteristics of older decedents by place of death: home, hospital and Social welfare facility, South Korea 2001-2014

Variable	Value	Home		Hospital		Social welfare facility		*p*-value*
		*N*	%	*N*	%	*N*	%	
Age, categorized	65-74	203,539	26.7	487,148	63.9	9032	1.2	<0.001
	75-84	307,165	31.4	577,655	59.1	23,392	2.4	
	85+	246,641	39.8	298,699	48.2	29,226	4.7	
Gender	Male	343,169	30.3	681,673	60.3	18,719	1.7	<0.001
	Female	414,176	33.8	681,829	55.6	42,931	3.5	
Marital status	Single	7468	23.1	21,114	65.3	1568	4.9	<0.001
	Married	307,704	29.7	634,106	61.3	13,980	1.4	
	Divorced	11,657	21.2	37,306	67.7	1810	3.3	
	Widowed	430,516	34.8	670,976	54.3	44,292	3.6	
Education level	Uneducated	387,858	40.9	463,485	48.9	30,256	3.2	<0.001
	Elementary	244,032	29.5	501,528	60.7	20,028	2.4	
	Middle/High school	103,678	22.6	309,134	67.3	9110	2.0	
	University and higher	21,777	17.6	89,355	72.0	2256	1.8	
Residential area	Rural	201,438	38.9	279,800	54.1	10,883	2.1	<0.001
	City	316,624	32.8	542,986	56.3	28,746	3.0	
	Metropolitan	147,200	27.9	338,537	64.2	9426	1.8	
	Capital	92,083	26.4	202,179	58.1	12,595	3.6	
Cancer cause	Cancer	122,162	20.8	438,733	74.6	5801	1.0	<0.001
	Non-cancer	635,183	35.9	924,769	52.3	55,849	3.2	
Cause of death	Cancer (C00-C97)	122,162	20.8	438,733	74.6	5801	1.0	<0.001
	Dementia (F00-F03)	19,221	36.7	26,124	49.9	3926	7.5	
	IHD (I20-I25)	28,449	25.6	69,234	62.4	2239	2.0	
	CVDs (I60-I69)	80,457	33.7	138,492	58.0	6044	2.5	
	COPD (J40-J47)	31,996	32.5	59,474	60.4	1538	1.6	
	Parkinson’s Disease (G20)	5147	24.1	13,759	64.3	1255	5.9	
	Alzheimer’s Disease (G30)	4585	17.0	19,687	72.9	1508	5.6	
	HIV (B20-B24)	25	15.3	132	81.0	2	1.2	
	Others	465,328	38.1	597,999	49.0	39,339	3.2	
Seasonal period	Spring	198,207	33.0	341,536	56.9	15,149	2.5	<0.001
	Summer	169,106	31.0	323,291	59.2	13,721	2.5	
	Autumn	186,527	31.5	345,483	58.3	15,914	2.7	
	Winter	203,505	32.9	353,192	57.0	16,866	2.7	

*p-value from Pearson’s Chi-squared test

Table 3 shows the results from logistic regression younger decedents exhibited a higher probability of dying in a hospital than the oldest age group (ORs for the 75-84 years age group of 1.28, 95% CI 1.27-1.29 and for the 65-74 years age group of 1.30, 95% CI 1.29-1.31). Females were more likely to die in a hospital than males (ORs 1.28, 95% CI 1.27-1.29). Among individuals that died from non-cancer causes, those of older age were more likely to die in a hospital. For other factors, including sex, marital status, education level, and residential area, similar results with the overall population were found between subjects divided according to cancer and non-cancer causes of deaths.

**Table 3:** Associated factors with hospital death (vs. home death) from univariate and multivariate logistic regression models

Variable	Value	Overall				By cause of death							
						Cancer				Non-cancer			
		Crude		Adjusted		Crude		Adjusted		Crude		Adjusted	
		ORs	95% CI	ORs	95% CI	ORs	95% CI	ORs	95% CI	ORs	95% CI	ORs	95% CI
		*N* = 2,120,847				*N* = 560,895				*N* = 1,559,952			
Age, categorized	85+	1.00		1.00		1.00		1.00		1.00		1.00	
	75-84	1.55	1.54-1.56	1.28	1.27-1.29	1.08	1.06-1.11	1.03	1.01-1.05	1.44	1.43-1.45	1.30	1.29-1.31
	65-74	1.98	1.96-1.99	1.30	1.29-1.31	1.21	1.19-1.24	1.05	1.03-1.07	1.69	1.67-1.70	1.33	1.32-1.34
Gender	Male	1.00		1.00		1.00		1.00		1.00		1.00	
	Female	0.83	0.82-0.84	1.28	1.27-1.29	1.17	0.16-1.19	1.45	1.43-1.48	0.88	0.87-0.88	1.24	1.23-1.25
Marital status	Married	1.00		1.00		1.00		1.00		1.00		1.00	
	Single	1.37	1.34-1.41	1.77	1.72-1.82	1.68	1.57-1.80	1.77	1.65-1.90	1.47	1.43-1.51	1.74	1.69-1.79
	Divorced	1.56	1.52-1.59	1.49	1.46-1.53	2.19	2.09-2.31	1.93	1.83-2.03	1.50-	1.47-1.54	1.39	1.35-1.42
	Widowed	0.76	0.74-0.76	1.03	1.02-1.04	1.08	1.07-1.10	1.38	1.11-1.16	0.81	0.81-0.82	1.01	*0.99-1.01*
Education level	Uneducated	1.00		1.00		1.00		1.00		1.00		1.00	
	Elementary	1.72	1.71-1.73	1.61	1.60-1.63	1.39	1.36-1.41	1.54	1.52-1.57	1.65	1.64-1.66	1.63	1.62-1.64
	Middle/High school	2.50	2.47-2.52	2.27	2.25-2.29	2.08	2.04-2.12	2.38	2.33-2.43	2.24	2.22-2.26	2.21	2.20-2.24
	University and higher	3.43	3.38-3.49	3.17	3.12-3.23	2.97	2.88-3.06	3.48	3.35-3.58	2.98	2.92-3.03	3.05	2.99-3.11
Residential area	Rural	1.00		1.00		1.00		1.00		1.00		1.00	
	City	1.23	1.23-1.24	1.13	1.13-1.14	1.39	1.37-1.42	1.25	1.23-1.28	1.19	1.18-1.20	1.10	1.09-1.11
	Metropolitan	1.66	1.64-1.67	1.46	1.45-1.48	1.86	1.83-1.90	1.59	1.56-1.62	1.62	1.60-1.63	1.43	1.42-1.44
	Capital	1.58	1.57-1.60	1.26	1.25-1.28	2.00	1.96-2.05	1.54	1.51-1.58	1.46	1.44-1.47	1.20	1.18-1.21
Cancer cause	Non-cancer (others)	1.00		1.00									
	Cancer (C00-C97)	2.47	2.45-2.48	2.18	2.17-2.20								
Seasonal period	Spring	1.00		1.00		1.00		1.00		1.00		1.00	
	Summer	1.12	1.10-1.12	1.09	1.08-1.09	1.14	1.12-1.16	1.14	1.12-1.16	1.08	1.07-1.09	1.07	1.06-1.08
	Autumn	1.07	1.07-1.08	1.06	1.06-1.07	1.12	1.10-1.14	1.11	1.09-1.13	1.05	1.05-1.06	1.05	1.04-1.06
	Winter	1.01	*0.99-1.02*	1.01	1.01-1.02	1.05	1.03-1.07	1.05	1.03-1.07	1.01	*0.99-1.01*	1.01	*0.99-1.01*

Abbreviations: *ORs* Odd Ratios, *CI* Confidence Interval. Italic numbers: not statistically significant results

**Fig. 2:**
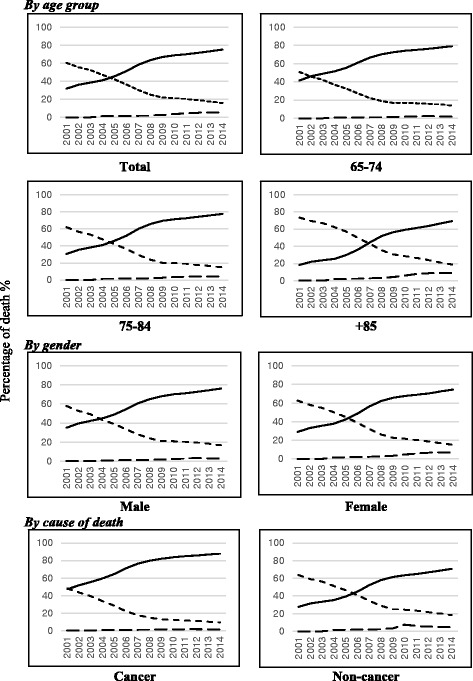
Trends in place of death in Korea elderly population, 2001-2014 by age group, gender and cancer/non-cancer cause of death. Trend of hospital death was illustrated by solid line, dotted line shows trend of home death and dashed line represents for trend of death in social welfare facilities

Figure 2 illustrates trends in the proportions of deaths at hospitals, home, and social and welfare facilities in South Korea from 2001 to 2014 according to age, sex, and causes of death. Overall, there was an upward trend in hospital deaths and a downward trend in deaths at home during this period, regardless of age, sex, and cause of death. Deaths at social and welfare facilities increased slightly and steadily, particularly after 2009. Hospital deaths started increasing earlier among the younger age group and those who died from cancer. There were no considerable differences in deaths at social and welfare facilities between males and females. Meanwhile, however, people who died after the age 85 of years or died from non-cancer causes tended to die more often in these facilities.

**Fig. 3:**
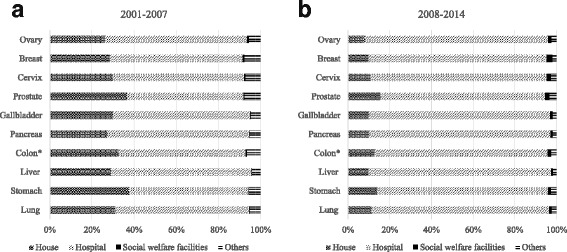
Difference in distribution of places of death by cancer sites between two periods: 2001-2007 and 2008-2014. Places of death include house, hospital, social welfare facilities and others. **a** represents for death cases from 2001 to 2007, **b** represents for death cases from 2008-2014. *Colon/rectal cancer

Figure 3 illustrates differences in the distribution of places of death according to cancer sites between two periods of time (2001-2007 and 2008-2014). For all cancer sites, compared with the first period, we noted an increase in the proportion of hospital deaths and a decrease in deaths at home. Compared to other cancers, the proportions of deaths at home were higher among those who died of prostate or stomach cancer (15.6% and 13.9% in 2008-2014 period).

## Discussion

Utilizing death data for a 14 year-period, we explored trends in places of death in South Korea, primarily focusing on hospital deaths. Deaths in a hospital gradually increased from 2001 to 2014. Together with a previous study on the same topic [18], we found that hospital deaths in South Korea have increased since 1993 and that increase therein has remained consistent over the last two decades. In addition to South Korea, greater institutionalization of dying has also been noted in other countries**.** Other Asian countries have documented similar shifts from deaths at home to hospital deaths, including Japan and Taiwan. In Japan, a significant reduction in home deaths was observed, from 82% to 13%, as a result of rapid of growth in health care services in the 1960s to 1980s [12, 21]. A study conducted for Taiwanese cancer patients also noted a decreasing trend in home deaths [22]. These patterns are in stark contrast to those in Western countries, including British [23], England [15], US [24, 25], and Canada [26]. Previous studies in those countries have documented decreases in deaths at a hospital and a shift toward deaths at home or nursing homes. Similar trends were observed in Belgium and Australia [17, 27]: a shift to out-of-hospital deaths, such as death in a nursing home (including residential and skilled nursing care home). In Belgium, from 1998 to 2007, hospital deaths decreased from 55.1% to 51.7%, and care home deaths rose from 18.3% to 22.6%. The study in Belgium predicted that continuation of these trends would result in a doubling of deaths in care homes and significant decreases in deaths at home and in hospitals by 2040 [17].

Rapid economic transformation has brought about significant changes in South Korea’s demographics, as well as other society-altering phenomena, such as urbanization, changes in living arrangements, and household structure. According to a report from the Korean Ministry of Health and Welfare in 2004, more than half of Korean older adults live alone and/or with only their spouse [28]. Furthermore, results of a survey by a government research institution estimated that the proportion of households with occupants living alone and/or with only their spouse would increase to over 70% in 2010 [29]. These alterations in household structure make it no longer possible to maintain traditional systems of family-based care for older people. Therefore, the vast majority of older people who are in need of care and assistance must be admitted to a hospital to receive appropriate care. We presume that this major demographic issue has contributed to the noted increases in deaths at a hospital.

One study in South Korea concluded that greater hospital bed availability has facilitated a greater odds of dying in a hospital [18]. Their analysis further suggested that hospital bed availability was more strongly associated with place of death than place of residence [18]. Since 1990, the objectives of the Korean health care system have changed with efforts to achieve universal coverage [30]: Policies throughout the 90s mainly targeted accessibility and universality; they now focus on efficiency, equity, and quality of care. As a result, health care resources have dramatically improved, facilitating enormous increases in hospital beds [31]. Universal health care has provided Koreans with better access to medical services, particularly hospital-based care, and we believe that this has, in turn, contributed to increases in hospital deaths. Meanwhile, however, although palliative care and long-term health care were introduced in South Korea in 2003 and 2008, respectively, health care systems in South Korea have been deemed to be ill equipped to cope with a rapidly aging population [19]. Facilities, as well as health care services, for end-of-life care or palliative care have remained under-developed and insufficient [19]. Consequently, patients requiring these services have been found to occupy acute care beds in hospitals, potentially leading to the increasing number of deaths in hospitals [30].

Knowledge of determinants influencing place of death can be helpful to developing public health strategies targeting end-of-life care. Results from our study indicated that age, sex, education level, marital status, residence area, and causes of death are significantly associated with place of death. In our study, we found that increasing age was consistently a predictor of a reduced likelihood of dying in a hospital. This finding is consistent other studies [3, 18, 27]. One study conducted in Western Australia found that, beyond the age of 55 years, the rate of people who died in the hospital rises; however, after 85 years, people, especially females, tended to die more often in their place of residence [32]. Inconsistent results were found in a study of Canadians [33], wherein, although age also was a strong determinant of place of death, younger people were more likely to die at home and older people were more likely to die in extended care facilities.

Herein, we noted a striking discrepancy in places of death between less and more highly educated individuals. In our study, higher education was consistently a strong predictor of a higher likelihood of dying in a hospital and was consistent with previous investigations in South Korea [18, 34]. In some European countries, however, where home-based care or other types of end-of-life care are more developed, contrasting patterns in places of death according to education status have been observed: people with higher education less often die in a hospital [35]. Offering explanation thereon, the authors posited that education may affect differences in treatment or care preferences and differences in an individual’s capability to express those preferences. Highly educated people generally can communicate better with physicians and have well thought out plans on their dying trajectories. Moreover, in developed countries, less education is frequently associated with fewer financial resources, which consequently result in inaccessibility to expensive in-home care services. Therefore, institutional care and dying in a hospital is plausibly more frequent among lower educated people. Some studies, such as one in Germany, however, were unable to find any clear link between educational background and place of death [36].

Our analyses highlighted relationships between marital status and where people die similar to those in previous studies [18, 35–37]. We found that decedents who were either single, divorced, or widowed for both sexes were more likely to die in a hospital than married decedents. We also discovered that older Korean adults living in more urbanized areas are more likely to die in a hospital, especially those living in metropolitan areas. Similarly, previous studies have reported that modern metropolitan cities have persistently high rates of hospital death [3, 18, 33, 35]. The influence of urbanization on places of death could be associated with better accessibility to care facilities and higher hospital bed availability in more urban areas.

In the present study, places of death differed substantially between older adults who died from cancer and those who died from other causes. In particular, compared to non-cancer, hospital deaths were appreciably more common among cancer decedents. This finding is congruent with previous investigations [18, 21, 35]. Terminal cancer patients frequently require palliative care in their last few months, and long hospital stays may increase the likelihood of dying in a hospital. In addition, as end-of-life care at nursing homes or in-home is not well developed in South Korea, cancer patients in South Korea typically die in hospitals. Palliative care is a relatively new focus of the health care in South Korea. Currently, palliative care in South Korea remains exclusively available to only people with cancer [30]. We suspect that this is the reason for the greater number of hospital deaths among older adults dying from cancer, compared to those dying from non-cancer diseases, in our study. Moreover, only some medical aspects and symptom management services are reimbursed under the National Health Insurance system. Therefore, patients in need of pain relief and who face difficulties with accessing specialist palliative care services must occupy acute care beds in hospitals. As a result, hospitals in South Korea have begun to offer palliative care services as a component of integrated treatment [30]. Meanwhile, however, research in other countries has concluded that cancer patients are more likely to die at home or other places than a hospital [33, 35]. Therein, the authors hypothesized that, since the disease course of cancer is more predictable and confers longer survival after diagnosis, cancer patients may have more time to prepare for approaching death; therefore, they can better choose where they want to die [36]. This discrepancy might reflect better development of other types of end-of-life services in some European countries [5].

This study analyzed of a large unique dataset to outline variations in places of death among older adults in South Korea. Analyzing datasets for 14 years, we conducted a comprehensive analysis through which to identify trends and distributions in places of death. Other strengths of this study include the high quality of the datasets with few missing cases and the population-based nature of the datasets. This study does have some limitations that warrant consideration. The use of death certificate data is inherently limited. First, as it is an administrative dataset, we cannot take into account several important factors that may have an impact on where people eventually die; these include medically related factors, such as characteristics of the course of the disease and dying process, and information on the decedent’s income and social support. Death certificate data also lack information on the patients’ preferences on their place of dying. Second, statistical patterns from this quantitative research does not allow us to draw an absolute conclusion about the choices, behaviors, or attitudes underlying those patterns. Another noteworthy point is that, since the South Korean government has implemented long-term care insurance on a nationwide scale, the numbers of both general and long-term care hospitals have increased dramatically recently. However, in the death registration database, long-term care hospitals and palliative care are coded together with general and other kinds of hospitals. Due to this limitation, we could not explore the distribution of deaths in this particular setting.

## Conclusion

Utilizing Death Registration Database, this study outlined high quality empirical data on where older adults in South Korea died, together with information on factors associated therewith. Our results showed that there was a consistent increasing trend in hospital deaths in South Korea from 2001 to 2014. Therefore, we suggested that more sufficient resources and facilities should be provided to fulfill the end-of-life care preferences and needs of Korea population. Furthermore, forward future planning for palliative care should consider individuals dying from conditions with severely life-limiting symptoms other than cancer, as well as expanding the scope of services and coverage. To avoid unnecessary admissions to hospitals during the last few months of life, enhancing non-hospital based palliative care centers, such as home visits or day-care centers, could be a potential solution.
